# Importance of Genetic Studies in Consanguineous Populations for the Characterization of Novel Human Gene Functions

**DOI:** 10.1111/ahg.12150

**Published:** 2016-03-22

**Authors:** A. Mesut Erzurumluoglu, Hashem A. Shihab, Santiago Rodriguez, Tom R. Gaunt, Ian N.M. Day

**Affiliations:** ^1^ Bristol Genetic Epidemiology Laboratories (BGEL), School of Social and Community MedicineUniversity of BristolBristolUK; ^2^ MRC Integrative Epidemiology Unit (IEU), School of Social and Community MedicineUniversity of BristolBristolUK; ^3^ Genetic Epidemiology Group, Department of Health SciencesUniversity of LeicesterLeicesterUK

**Keywords:** Consanguineous populations, gene function, autozygosity, Mendelian disease, complex disease

## Abstract

Consanguineous offspring have elevated levels of homozygosity. Autozygous stretches within their genome are likely to harbour loss of function (LoF) mutations which will lead to complete inactivation or dysfunction of genes. Studying consanguineous offspring with clinical phenotypes has been very useful for identifying disease causal mutations. However, at present, most of the genes in the human genome have no disorder associated with them or have unknown function. This is presumably mostly due to the fact that homozygous LoF variants are not observed in outbred populations which are the main focus of large sequencing projects. However, another reason may be that many genes in the genome—even when completely “knocked out,” do not cause a distinct or defined phenotype. Here, we discuss the benefits and implications of studying consanguineous populations, as opposed to the traditional approach of analysing a subset of consanguineous families or individuals with disease. We suggest that studying consanguineous populations “as a whole” can speed up the characterisation of novel gene functions as well as indicating nonessential genes and/or regions in the human genome. We also suggest designing a single nucleotide variant (SNV) array to make the process more efficient.

## Introduction

Autozygosity mapping has proven to be a powerful technique for unearthing autosomal recessive disease causal mutations in consanguineous offspring (Carr et al., 2013). However, even after decades of studying consanguineous families with disorders, many genes in the genome still do not have a clinical phenotype associated with them. This could be because most genes in the genome do not cause a clinical/defined phenotype (e.g., early‐onset disorders), which have traditionally been the main focus of genetic association (and linkage) studies. Therefore, a paradigm shift is required in order to discover the function of the remaining genes. All genes need to be observed when completely “knocked out” [i.e., rendered completely dysfunctional or inactivated by homozygous loss of function (LoF) mutations], similar to reverse genetics studies carried out in model organisms in order to better understand the function of genes by inactivating the gene and studying the resulting phenotype. The study of consanguineous unions is the closest human equivalent to these types of studies. However, it is clear that all the answers do not lie only in the study of consanguineous offspring with disorders. Consanguineous offspring without distinct clinical phenotypes should also be analysed to observe which genes harbour homozygous LoF mutations. This can then shed light on the function of these genes since these individuals can be followed up through cohort studies to observe any long‐term effects, and by molecular studies to observe any subtle differences such as changes in the expression of other genes. It may be that the mutated gene is dispensable, and thus is on its way to becoming a pseudogene. “Knocking out” certain genes may even have protective effects against certain disorders or diseases. In this paper, our aims are to put forward some arguments for undertaking a gene‐centric approach rather than the traditional disorder‐based approach when analysing consanguineous populations. We have also made suggestions about which consanguineous populations are most suitable for analysis.

## Studying Consanguineous Individuals and Populations

**Figure 1:**
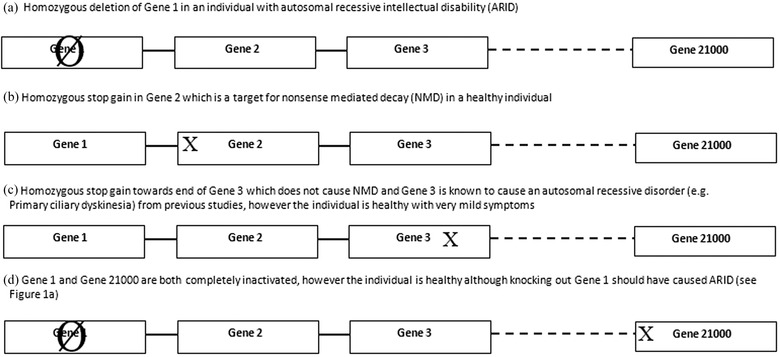
Examples of inferences to be gained from autozygous regions in consanguineous offspring. (a) Homozygous LoF mutations in gene 1 causes ARID, (b) Gene 2 is likely to be a nonessential gene (i.e., dispensable). The subject should be followed up for late‐onset effects or via deeper phenotyping. (c) Although gene 3 can cause primary ciliary dyskinesia (PCD), the coding region from the stop gain to the end of the exon is not essential for correct functioning of the gene, hence the unaffected subject (NB: mutation is not a target for NMD). (d) Although LoF mutations in gene 1 cause ARID, concurrent inactivation of gene 21000 (arbitrarily chosen number) due to NMD masks disease phenotypes indicating interaction between the two genes products in the causal pathway (e.g., gain of function mutation at gene 1 could become dysfunctional by mutation at gene 21000). X: Stop gain. Ø: Deletion/inactivation of whole gene. Position of stop gain within genes is for illustration purposes. This is not an exhaustive list of all the possible inferences which could be gained from studying consanguineous populations (e.g., identifying dispensable regions, proxy molecular diagnoses (see Erzurumluoglu et al. 2015b for details on the latter).

There are over 7500 disorders with a known or suspected Mendelian basis, and 4473 have had their molecular basis determined (from Online Mendelian Inheritance in Man, OMIM; statistics true as of 23‐06‐15) (Clamp et al., 2007; Online Mendelian Inheritance in Man, 2013). However, over half of these are caused by autosomal dominant mutations (thus causing autosomal dominant disorders), and the rest are autosomal recessive or X‐linked. Thus, given the estimation of ∼20,000 genes in the human genome, we have not observed the homozygous effects of mutations causing LoF for over 10,000 genes (up to 15,000, excluding ∼2000 genes located in sex chromosomes and the commonly “knocked out” autosomal genes such as olfactory receptor genes). This is presumably mainly due to the fact that most current studies apply selection on disease phenotypes in humans. Our “phenotypic ascertainment” claim is backed up by the high proportion of autosomal dominant disorder causal mutations identified in comparison to autosomal recessive disorder causal ones. The Hardy–Weinberg (H‐W) equation clearly states that in an outbred population, the proportion of heterozygotes (i.e., 2pq) will be considerably higher than homozygotes of the causal variant (2pq > q^2^, 2q for very low q); thus, it is no surprise that when one ascertains for disease phenotypes, more autosomal dominant mutations will be identified (Mayo, 2008). We therefore suggest that sampling individuals randomly from a consanguineous population, as opposed to only targeting families with disease, will avoid this ascertainment bias and identify more homozygous LoF mutations in most, if not all genes, given sufficient sample sizes. See Figure 1 for examples of inferences which could be made from analysing autozygous regions in consanguineous offspring.

There are regions and subregions in the world where consanguineous unions are preferred for various socioeconomic reasons, with consanguinity levels reaching as high as 70% (Hashmi, 1997). Despite the importance of consanguinity to genetic research, most of these populations are far from being thoroughly researched in genetic and sociological terms (Bittles, 2001; Bittles & Black, 2010). Larger sequencing projects are required to make full use of these populations and such studies would serve human genetics immensely.

### Effects of Consanguinity on Mendelian Disease

Very rare recessive mutations are predicted to be present in every population but since they rarely achieve homozygosity in outbreeding populations, they are mostly passed onto future generations silently. However, unions amongst relatives dramatically increase the probability of being homozygous at any genetic locus in the offspring (Fig. S5). This is why very rare autosomal recessive disorders are predominantly observed in regions where there are high levels of endogamy or in families where the parents are closely related. Studying these populations will increase considerably the number of homozygous gene knockouts identified.

We recently published a review on how best to identify highly penetrant disease causal mutations which provides an analysis schema to make the process more efficient and reliable (Erzurumluoglu et al., 2015a). The schema can also apply to monogenic forms of common‐complex disorders, for example, mutations in the leptin gene and obesity (Montague et al., 1997).

### Effects of Consanguinity on Common Complex Diseases

The significance of human consanguinity on complex disorders *per se* is largely unknown and the literature on the subject matter is inconclusive (Bittles & Black, 2010). The role played by consanguinity on complex disorders is likely to vary depending on which model explains the genetic basis (i.e., the true underlying biology) of the complex disorder in question. Examples of such models include the infinitesimal model, the rare allele model and the broad sense heritability model (see Gibson, 2011 for details). Consanguinity would be expected to have a greater influence on a complex disease if the rare variant model is used to explain the aetiology of the disorder. A varying but lesser effect would be expected with the broad sense heritability model in accordance with the influence of environmental factors such as epigenetic factors and gene‐environment interactions, on the disorder. With the infinitesimal model, one would predict the effect of consanguinity *per se* on the disorder to be that of a very small effect brought about only due to higher levels of homozygosity in consanguineous offspring (i.e., the effect of the minor allele is doubled in homozygotes compared to heterozygotes). This is because approximately 15/16 (93.75%) of the genome remains relatively “outbred” even in the offspring of first cousins.

In order to reliably deduce the role of consanguinity in complex disorders, many environmental factors have to be considered as well as genetics and health‐related factors (Hamamy et al., 2011). The effect of consanguinity on complex disorders *per se* cannot be reliably analysed through simple consanguineous versus nonconsanguineous population comparisons, as has been done previously, and many factors need to be controlled for (see Fig. S4) (Bittles & Black, 2010).

### Natural Human Gene Knockouts in Consanguineous Populations

**Figure 2:**
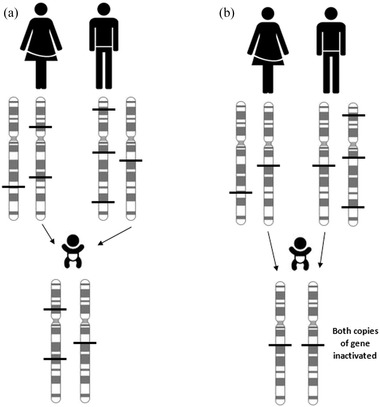
Example of difference between union of (a) unrelated and (b) related individuals. Although everyone possesses rare LoF mutations within their genome, they are likely to be unique to their family (or themselves). Therefore, the offspring of unrelated individuals have an almost zero probability of being homozygous for these variants. Since related individuals will have a fairly recent common ancestor, their ancestors’ LoF mutations will be passed on and there is on average a 6.25% chance of these mutations to be in a homozygous (or more correctly, autozygous) state in the offspring of first cousins. Thick black lines represent LoF mutations. The figure has been simplified for clarity (e.g., does not include recombination events).

Empirical studies show that each individual, depending on ancestry, possesses between 10 and 20 rare mutations which could introduce premature nonsense codons (Ng, 2009), although these are mostly present in the heterozygous state. The number of mutations causing LoF will be increased with the addition of rare frameshifting indels [found to be between 8 and 17 indels, depending on ancestry (Ng, 2009)], rare functionally disruptive missense mutations and splice site acceptor or donor mutations (between 40 and 60 and ≤2, respectively, internal data from nine whole‐exome sequenced individuals—unpublished data). Due to the elevated probability of an allele being homozygous in a consanguineous individual, it is likely that at least one gene will be completely dysfunctional or inactivated as a result of these rare mutations (Fig. 2).

Knockout studies in model organisms are well established and have hugely facilitated our understanding of our own genome and the biological pathways which connect some of these genes. However, where not backed up by human observational studies, animal knockouts can be misleading as the underlying mechanism may be different in the model organism or the gene may have a different (or other acquired) function(s). In addition, some human genes lack homologues in the commonly analysed model organisms; some, termed “orphan” genes, may even have no homologues at all (Miklos et al., 1997) which is another limitation of these gene knockout studies (Tautz & Domazet‐Loso, 2011). Therefore, candidate genes derived from model organism “knockouts” cannot be directly translated to a human model until the same phenotype is also observed in humans.

However, sampling randomly from a consanguineous population will enable the identification of natural human knockouts, enabling the identification of nonessential genes, genes which cause late onset disorders (for example, highly penetrant mutations in certain genes causing certain cancers) and embryo loss, alongside those genes causing Mendelian disease. In this sense, studies of consanguineous populations can be classified as examples of a “*quasi*‐reverse genetics” study (QRG), with direction of study being “genotype to phenotype.” Simply put, those genes which have been inactivated in a consanguineous individual can be determined initially using whole‐exome sequencing (WES) [or whole‐genome sequencing (WGS) where feasible], then the short‐term and long‐term effects, if any, can be observed (see Fig. 1 for some examples).

#### Frequency of natural gene knockouts

To understand clearly what the analysis of consanguineous collections can offer for human genetics, a comparison between an outbred and a consanguineous collection must be made (Tables 1 and 2). Consider a hypothetical outbred population in H‐W equilibrium for a wild‐type (and common) allele of frequency p and an inactivating allele of frequency q (i.e., the rare allele), where p + q = 1. Homozygotes for the rare allele will be found at frequency q^2^. However, in a consanguineous collection with a certain $\bar{F}$ (average inbreeding coefficient), an allele with frequency q will be expected to be in a homozygous state at a frequency of approximately $\bar{F}$ × q (i.e., the overall likelihood of autozygosity for any given allele multiplied by the frequency of the rare allele in the population—see row 1 of Tables 1 and 2 for more accurate comparisons).

**Table 1:** Comparison between collections of outbred offspring versus offspring of first cousins. Offspring of first cousins are expected to have an *F* value of 0.0625. For example, for a disorder such as (autosomal recessive) familial hypercholesterolemia with a global prevalence of 1 in a million according to the H‐W equation, one would estimate the frequency of the causal allele (i.e., q) to be 1 in 1000. However, in a consanguineous population, this figure will be inflated approximately 60‐fold to around 1 in 16,000. MAF: Minor allele frequency

		3. Heterozygote frequency	4. Homozygote frequency	5. Frequency of	6. Relative odds of
		in outbreeding	in outbreeding	homozygotes (of q) in First	finding homozygotes
1. Row	2. MAF (q)	population (2pq)	population (q^2^)	offspring (q^2^+(1‐q)qF)	((1+F(1‐q))/q)
1	0.1	0.18	0.01 (1/100)	0.015625	×1.6
2	0.0316	0.0432	∼0.001	0.00291259	×2.9
3	0.01	0.018	0.0001 (1 in 10000)	∼0.000725	×7.2
4	0.00316	0.00432	∼0.00001	∼0.0002075	×20.7
5	0.001	0.0018	0.000001 (1 in a million)	∼0.0000635	×63.5
6	0.000316	0.000432	∼0.0000001	∼0.00001985	×198.5
7	0.0001	0.00018	0.00000001	∼0.00000626	×626
8	0.0000316	0.0000432	∼0.000000001	∼0.00000198	×1978

**Table 2:** Comparison between collections of outbred offspring versus offspring of uncle–niece unions (or double first cousins). Offspring of first cousins are expected to have an *F* value of 0.125, whereas the expected *F* value for the offspring of outbred individuals is (very near) zero. MAF: Minor allele frequency

		3. Heterozygote frequency	4. Homozygote frequency in	5. Frequency of	6. Relative odds of
		in outbreeding	in outbreeding	homozygotes (of q) in offspring of	finding homozygotes
1. Row	2. MAF (q)	population (2pq)	population (q^2^)	uncle‐niece unions (q^2^+(1‐q)qF)	((1+F(1‐q))/q)
1	0.1	0.18	0.01 (1/100)	0.02125	×2.1
2	0.0316	0.0432	∼0.001	0.00482518	×4.8
3	0.01	0.018	0.0001 (1 in 10000)	0.0013375	×13.4
4	0.00316	0.00432	∼0.00001	0.0004049605	×40.5
5	0.001	0.0018	0.000001 (1 in a million)	∼0.000126	×126
6	0.000316	0.000432	∼0.0000001	∼0.0000396	×396
7	0.0001	0.00018	0.00000001	∼0.00001251	×1251
8	0.0000316	0.0000432	∼0.000000001	∼0.000003951	×3951

Tables 1 and 2 illustrate the differences in homozygote frequencies between outbred and consanguineous populations for alleles with a range of different frequencies. It is clear that there is a higher probability of observing a homozygote for a rare inactivating variant in a consanguineous collection (see column 6 of Tables 1 and 2). In contrast, there is a negligible chance, even with a large sample size, of observing a homozygote for a rare allele in a randomly breeding population. See Figures S1 and S2 for a comparison of alleles with minor allele frequencies (MAFs) of 0.1 and 0.001 in consanguineous populations.

Our base‐by‐base permutation analysis estimates that there are approximately 4.5 million potential stop‐gains, approximately 78 million missense mutations (with over 30 million predicted to be deleterious by SIFT and Polyphen‐2, and over 10 million by FATHMM) and approximately 0.5 million stop‐losses, as well as presumably thousands of essential splice site donor or acceptor variants to be observed in the human genome (see Table S1 for details) (Ng & Henikoff, 2003; Adzhubei et al., 2010; Liu et al., 2013; Shihab et al., 2013); and observing a sufficient number of these (i.e., at least one per each gene) in the homozygous state can only be feasible in consanguineous collections (see Tables 1 and 2).

#### Calculating the expected number of complete gene inactivations

Consanguineous populations are not well represented in large sequencing projects which are biased towards Western and/or Far Eastern countries, and this will cause unique and clinically relevant alleles present in these populations to be missed. Therefore, carrying out WES of consanguineous populations will allow identification of these unique alleles and, more importantly, in the homozygous state. A DNA bank of WES data from 10,000 participants who are offspring of consanguineous unions equal to or closer than first cousins would represent a resource of thousands of different combinations of gene inactivations in unrelated individuals (Equation 1.)

Total Ginactive=G¯xF¯xN

Equation 1 Calculating expected number complete gene inactivations (i.e., Total G_inactive_) in a consanguineous collection. $\bar{G}$: Average number of alleles causing LoF in individuals within a certain population/sample, $\bar{F}$: Average inbreeding coefficient of the database, N: Number of participants.

Using the equation above, the number of gene inactivations expected from such a collection can be calculated. For example, one would expect between 18 and 37 gene inactivations in any individual depending on their ancestry [adding together the figures of 10–20 rare stop gains and 8–17 frameshifting indels from (Ng, 2009)]. This would then be multiplied by the probability that any allele will be autozygous in the dataset, which will be 6.25% (i.e., 1/16) for a collection comprising mostly of offspring of first cousins (and 12.5% for a collection of offspring of uncle–niece unions and/or double first cousins) and the number of participating individuals, which will be arbitrarily chosen to be 10,000. Thus, one would expect between 11,000 and 24,000 (11,250–23,125 to be more exact using this example) complete gene inactivations caused by rare mutations in a collection consisting entirely of offspring of first cousins. This notable figure will be boosted with the addition of the offspring of uncle–niece and double first cousin unions which will increase the average inbreeding coefficient, while structural variation, LoF missense and splice‐site mutations will add considerably to the number of completely dysfunctional genes [MacArthur et al. predict this figure to be 100 LoF variants in healthy human genomes (MacArthur & Tyler‐Smith, 2010; MacArthur et al., 2012)]. Furthermore, homozygous stop gains which do not cause nonsense mediated decay (NMD) in clinically unaffected individuals can indicate exons which are not essential for gene function and vice versa, which can point to regions which are essential for development of disease in clinically affected individuals.

#### Possible way forward

Using a brute force approach to WES or WGS of as many consanguineous offspring as possible is presumably not going to be cost efficient as WES is still prohibitively expensive for very large‐scale sequencing studies, and many of the offspring will not harbour any “distinct” LoF variants in the homozygous state. There is also a lack of consensus as to what defines a “LoF” variant. Mostly, rare coding mutations which pass a certain arbitrarily chosen threshold for conservation, or which are predicted to be “deleterious” by a certain tool, are being clustered under the name “LoF.” However, where these variants are not followed up by functional studies such as gene expression studies, the evidence for the variant being “LoF” is usually very low and unconvincing.

Therefore, we propose that a SNP array containing probes for (i) all possible NMD causing stop gains and (ii) all other known LoF and/or disease causal mutations may be designed and used to screen as many consanguineous offspring as possible. Homozygous stop gains which are targets for NMD [i.e., in the 5′ end of the gene transcript and with >55bp remaining in the penultimate exon (Khajavi et al., 2006)] are highly likely to be LoF variants. Searching for these variants in a cost‐effective manner is bound to increase the number of homozygous “knockouts” identified in consanguineous populations. Such an SNP array would be better designed with expertise from different areas within the genetics field, including model organisms and public databases. Additionally, all possible mutations with a CADD score of over 50 (arbitrarily chosen here, representing the top 0.001% of predicted deleterious variants) (Kircher et al., 2014) and/or predicted deleterious by FATHMM‐MKL (arbitrarily chosen here, score of ≥0.98) (Shihab et al., 2015) could be added to the SNP array to validate the predictive power of these and similar tools.

Compared to the traditional approach of using SNP arrays to identify the autozygome of an individual followed by sequencing of these regions, the SNP array approach proposed here has several advantages. The SNP array would additionally identify variants which are homozygous as a result of endogamy and chance, whilst the traditional approach will only identify variants in the autozygome, excluding very short autozygous regions which are not identified; the SNP array would also identify variants in these regions. Furthermore, when carried out on a larger scale, identifying the autozygome for each individual and then designing primers to sequence these regions would become an unfeasible task. Such a SNP array is likely to serve the purposes of this type of study, as the power to detect novel homozygous LoF mutations will be directly proportional to the sample size. Once the feasibility and the efficiency of the array approach are confirmed, similar studies could be carried out in isolated and/or endogamous populations to search for more novel LoF variants in the homozygous state.

Given the very low costs of SNP arrays compared to WES (or WGS), there is greater scope for identifying “true” LoF variants with the former approach as the sample sizes will be much larger for the same costs . However, we must stress that we are not comparing WES/WGS with the SNP array approach proposed here *per se*, but rather we are comparing the two approaches in terms of characterising more novel and homozygous gene knockouts for similar costs.

#### Suitable populations for QRG studies

Many isolated populations and/or endogamous populations with small population sizes have been analysed, including the Croatian island populations, the Amish, the Icelanders and the Druze (Rudan et al., 2003). These studies have been useful in identifying dozens of founder mutations which cause disease (Norio, 2003). However, highly consanguineous populations which have a large population with a considerable amount of recent migration (thus with a rich gene pool) are likely to harbour considerably more LoF mutations in comparison to these isolated populations. Carrying out genetic studies in appropriately chosen populations will contribute greatly to our understanding of the function of many genes within the genome. A few suitable populations are discussed below.

##### City of Riyadh, Saudi Arabia

Located at the centre of the Arabian Peninsula and being the capital as well as the largest city of the Kingdom of Saudi Arabia (KSA), Riyadh has an ever increasing population size, with current estimates reporting an urban population figure of over 4 million. However, early in the twentieth century, the city's population was a mere 27,000 (Kim, 2013). This dramatic increase in population is due to three very influential factors: large family sizes (the average size is above six for Saudi families and approximately five for non‐Saudi families), rapid economic growth and immigration (for example, of Asians from Pakistan and India, and Arabs from Yemen and Egypt). Tens of thousands of mainly non‐Saudi rural dwellers still continue to migrate to the city of Riyadh each year (Kim, 2013). This influx of families from around the Arabian Peninsula translates into a very rich gene pool, important for the above‐mentioned reasons. Furthermore, Riyadh could be called a mecca for consanguinity with over 50% of total marriages being consanguineous, including a 30–40% first cousin marriage rate (α = 0.023) (Al Husain & Al Bunyan, 1997; El‐Mouzan et al., 2007; Bittles & Black, 2010). Within‐family marriages are a deep rooted tradition; thus, observed autozygosity is likely to be higher than standard estimates (meaning higher probability for an allele to be homozygous), which is another advantage of carrying out genetic analyses in Riyadh (Fig. S3). The quality of life is high in Riyadh, with access to advanced medical care and good communication services, and the country is relatively stable politically, economically and geographically compared other countries in the region (Kim, 2013). The King Saud University which is the leading university in the Arab world (according to the QS World University Rankings 2013, available at http://www.topuniversities.com/university‐rankings/world‐university‐rankings) and the King Faisal Specialist Hospital and Research Centre with its established centre for consanguinity studies are also located in Riyadh, both of which are important for possible collaboration. The initiation of the Saudi Human Genome Project (http://shgp.kacst.edu.sa/site/) is also an important platform for collaborative opportunities.

For a more comprehensive review on the genetic studies carried out in the KSA and the infrastructure that is available, see Alkuraya (2014).

##### Andhra Pradesh and Karnataka, India

Located in the South Eastern part of India, Andhra Pradesh has a population size of over 84 million (Chandrasekhara Rao, 2013). The city has a highly diverse population with many languages spoken from Telugu (the language of Andhra people) and Urdu (the language of Pakistanis) to Hindi (the language of modern day Indians) and Tamil (the language of Dravidian Indians). Next to Andhra Pradesh is Karnataka with also a diverse population of size of over 61 million. The Hindu societies in Andhra Pradesh and Karnataka show a remarkable contrast in the rate of consanguinity compared to other parts of India, where the overall rates have been low and/or diminishing (Bittles & Black, 2010; Chandrasekhara Rao, 2013). In particular, the rates of uncle–niece marriages reach as high as 20% of total Hindu marriages in Karnataka and approximately 5% in Andhra Pradesh; this is an important feature for genetic studies as their offspring are expected to be homozygous (i.e., autozygous) for 12.5% of their genome (Bittles, 2001; Kumar, 2004). In addition, health facilities have improved greatly in both cities due to continuous government funding (Chandrasekhara Rao, 2013). Average family sizes are also higher compared to European families (2.6 in India according to the Population Reference Bureau http://www.prb.org/). Consanguinity rates in Andhra Pradesh, Karnataka and Tamil Nadu are 30.8% (α = 0.0212), 29.7% (α = 0.018) and 38.2% (α = 0.026), respectively (Bittles, 2012).

##### Pakistan

Pakistan as a whole has a very high rate of consanguinity (over 40%) (Hamamy et al., 2011). However, it may not be feasible for QRG studies at present, not due to genetic and/or clinical factors but for political reasons such as periods of military rule, conflicts with India, and corruption (Nelson, 2009). With a population of over 180 million individuals and judging by the amount of infant/childhood deaths and autosomal recessive disorders in the Pakistani population living in the UK (especially the city of Bradford) (Sheridan et al., 2013), large‐scale studies carried out in Pakistan are likely to uncover many complete gene inactivations. Average family sizes are much higher compared to European families (3.6 in Pakistan according to the Population Reference Bureau).

##### Others

It may not be feasible to carry out large‐scale studies in many cities at once, thus small‐scale collaborations could be initiated in other populations where consanguinity rates are high. These include the Bedouin tribes/communities of the Arabian Peninsula [for example, in Oman, consanguinity rates reach as high as 50% (Islam, 2012)], certain populations in Bangladesh and some, mostly unexplored and endogamous, tribes of Africa. Such collaborations would no doubt increase the mutational spectrum identified by QRG studies.

Examples of consanguinity rates in other areas include:

Turkey (mostly in the East): 20.1% (α = 0.011) (Tuncbilek & Koc, 1994), Sudan: 52% (α = 0.0302) (Saha & El Sheikh, 1988), Jordan: 58.1% (α = 0.036—assuming all first cousin marriage) (Sueyoshi & Ohtsuka, 2003) and UAE: 50.5% (α = 0.0222) (al‐Gazali et al., 1997)

## Conclusions

Since many Mendelian disorders are rare and are caused by autosomal recessive alleles, more attention should be paid to regions where consanguinity is high; for a World map of consanguinity, see Bittles & Black (2010). However, we have also pointed out that selecting only for disease cases ignores genes without clinical relevance, as well as those which have subtle cellular effects or which may contribute to late onset disorders. For this reason, we also recommend sequencing and genotyping consanguineous individuals who do not show any clinical features early in life as well as those who do. It may even be the case that they harbour previously identified disease causal mutations but do not show any clinical signs as they simultaneously possess highly penetrant protective variants, for example, protecting against highly penetrant autosomal dominant mutations which interfere with other pathways. We have also suggested designing a SNP array to serve this purpose.

The traditional approach to consanguineous populations is to “cherry pick” families where a Mendelian disorder is segregating. Although this approach has yielded many disease causal loci, the effects of inactivation of both copies have still not been observed for most genes in the genome. This could be due to the above‐mentioned phenotypic ascertainment of families which prevents the identification of homozygous knockouts of other genes as they do not cause a Mendelian disorder, especially during childhood. Randomly sampling from a consanguineous population is bound to increase our understanding of the human genome by enabling characterisation of novel gene functions.

Previous studies have attempted to use nullizygous copy number variations (CNVs) and WES to identify dispensable DNA and genes in the genome (Khalak et al., 2012; Alsalem et al., 2013). These studies have served as small‐scale “proof‐of‐concept” experiments (with the traditional inclination towards disease phenotypes and/or other distinct traits), and therefore have largely gone unnoticed; thus, much larger studies with deep phenotyping are needed to understand the importance of consanguineous populations for human genetics. Very recently, two papers were made available in BioRxiv which carried out similar studies to the one proposed in this review (Narasimhan et al., 2015; Saleheen et al., 2015). Although the studies should be commended for their potential contributions to the literature, the criteria used by the authors to define “LoF” mutations are based on strong assumptions; and there is not much functional evidence provided by the authors that the variants identified do indeed cause LoF (i.e. complete loss of function) of the respective genes. The SNP chip array we propose here will concentrate on (homozygous) stop gains which are very likely targets for NMD (NB: the ones that should have been targeted but were not, should also be followed up by functional studies to understand the pathways involved), and therefore are very strong candidates for causing LoF of a gene, thus providing a more solid platform for characterising novel gene function. With the addition of known disease causal variants to the same SNP array, there is also the possibility of identifying protective variants which are highly penetrant with regard to their respective diseases or traits.

In this review, we have also provided a theoretical framework for calculating the expected number of genes with complete LoF taking into account variants of many types (i.e., all single nucleotide variation and indels). As opposed to the traditional approaches, this review underlines the importance of studying consanguineous populations as a whole. Additionally, we have identified suitable populations representing reliable stepping stones for the future direction of such analyses. The chosen populations, in order to be most effective, must have a rich gene pool as a result of mass migration and recent rapid population increase, while also being highly consanguineous and/or endogamous. Riyadh's population is a perfect example of this.

### Funding

Mesut Erzurumluoglu is a PhD student funded by the Medical Research Council (MRC UK). This work was supported by the Medical Research Council (MC_UU_12013/8 and G1000427).

### Conflict of Interest

None declared.

## Supporting information

Disclaimer: Supplementary materials have been peer‐reviewed but not copyedited.

**Table S1**: Potential LoF Mutations in the Human Genome.
**Figure S1**: Comparison between offspring of outbred individuals and first cousins using the example of an allele for which q = 0.1 (frequency of 1 in 10 in a population) and there are three unrelated homozygotes (i.e., AA) who marry into the family.
**Figure S2**: Comparison between offspring of outbred individuals and first cousins using the example of an allele for which q = 0.001 (frequency of 1 in thousand in a population).
**Figure S3**: Example of a complex pedigree with multiple consanguineous unions.
**Figure S4**: Factors influenced by consanguineous unions and/or by living in a highly consanguineous region.
**Figure S5**: Autozygosity mapping and consanguinity.
As a service to our authors and readers, this journal provides supporting information supplied by the authors. Such materials are peer‐reviewed and may be reorganised for online delivery, but are not copy‐edited or typeset. Technical support issues arising from supporting information (other than missing files) should be addressed to the authors.
