# Supplementary material for: Importance of Genetic Studies in Consanguineous Populations for the Characterization of Novel Human Gene Functions
Source: Ann Hum Genet. 2016 Mar 22;80(3):187–96. doi: 10.1111/ahg.12150 (PMC4949565; doi:10.1111/ahg.12150)
Supplement: Supplementary file 1 — Table S1: Potential LoF Mutations in the Human Genome. Figure S1: Comparison between offspring of outbred individuals and first cousins using the example of an allele for which q = 0.1 (frequency of 1 in 10 in a population) and there are three unrelated homozygotes (i.e., AA) who marry into the family. Figure S2: Comparison between offspring of outbred individuals and first cousins using the example of an allele for which q = 0.001 (frequency of 1 in thousand in a population). Figure S3: Example of a complex pedigree with multiple consanguineous unions. Figure S4: Factors influenced by consanguineous unions and/or by living in a highly consanguineous region. Figure S5: Autozygosity mapping and consanguinity. As a service to our authors and readers, this journal provides supporting information supplied by the authors. Such materials are peer‐reviewed and may be reorganised for online delivery, but are not copy‐edited or typeset. Technical support issues arising from supporting information (other than missing files) should be addressed to the authors. [file AHG-80-187-s001.pdf]

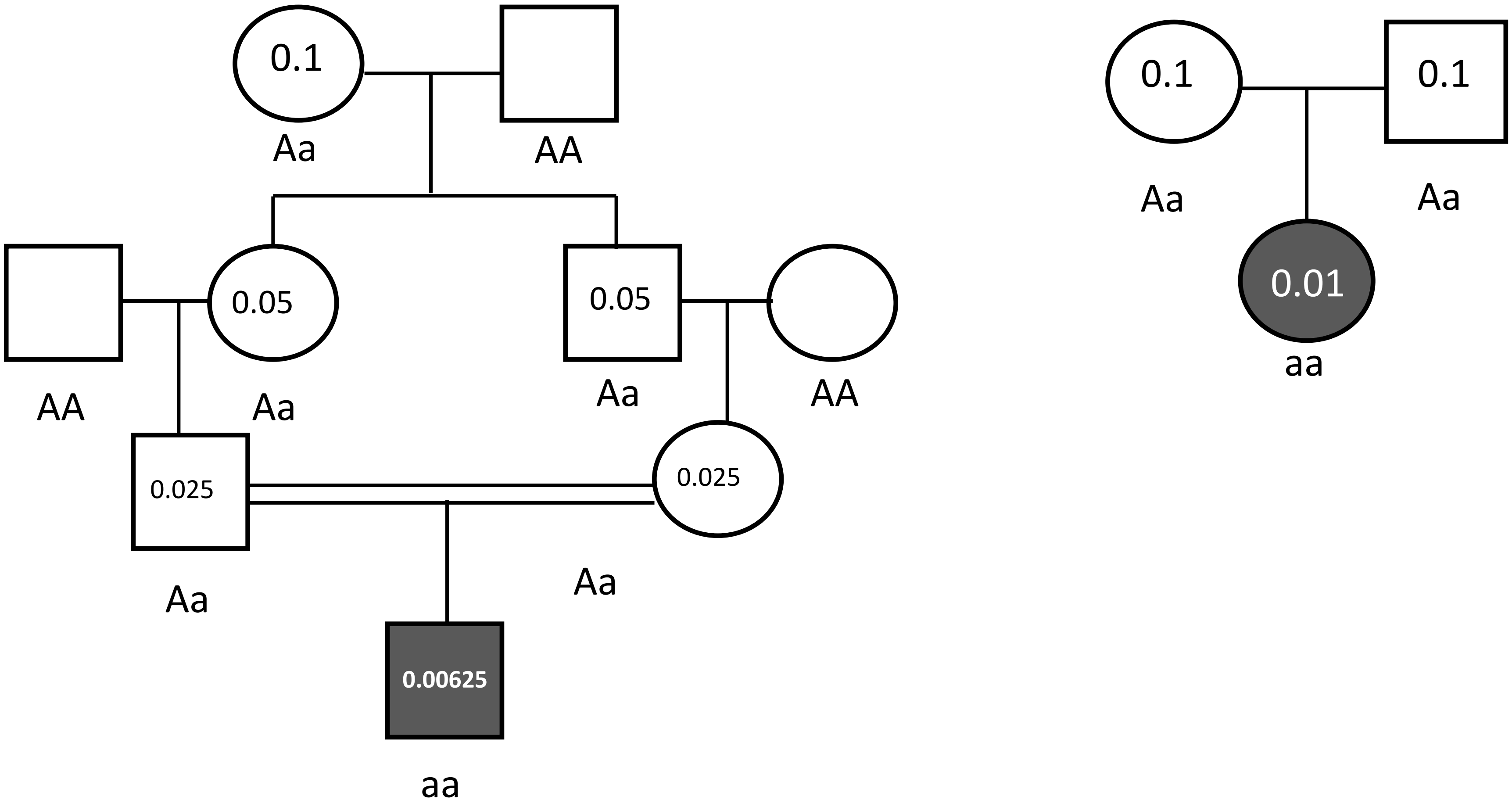

Supp. Figure S1 Comparison between offspring of outbred individuals and first cousins using the example of an allele for which q = 0.1 (frequency of 1 in ten in a population) and there are three unrelated homozygotes (i.e. AA) who marry into the family. In this scenario, when an allele is very common in a population (e.g. 0.1 as in this case), it is much more likely to find a homozygote in the offspring of outbred individuals compared to the offspring of first cousins. This is because of the lower probability of the LoF mutation travelling down the generations to meet its counterpart in her great-grandchildren. Due to purifying selection, we would not expect mutations causing LoF to be close to this frequency (see Supp. Figure S2). A: wild-type allele. a: LoF allele.

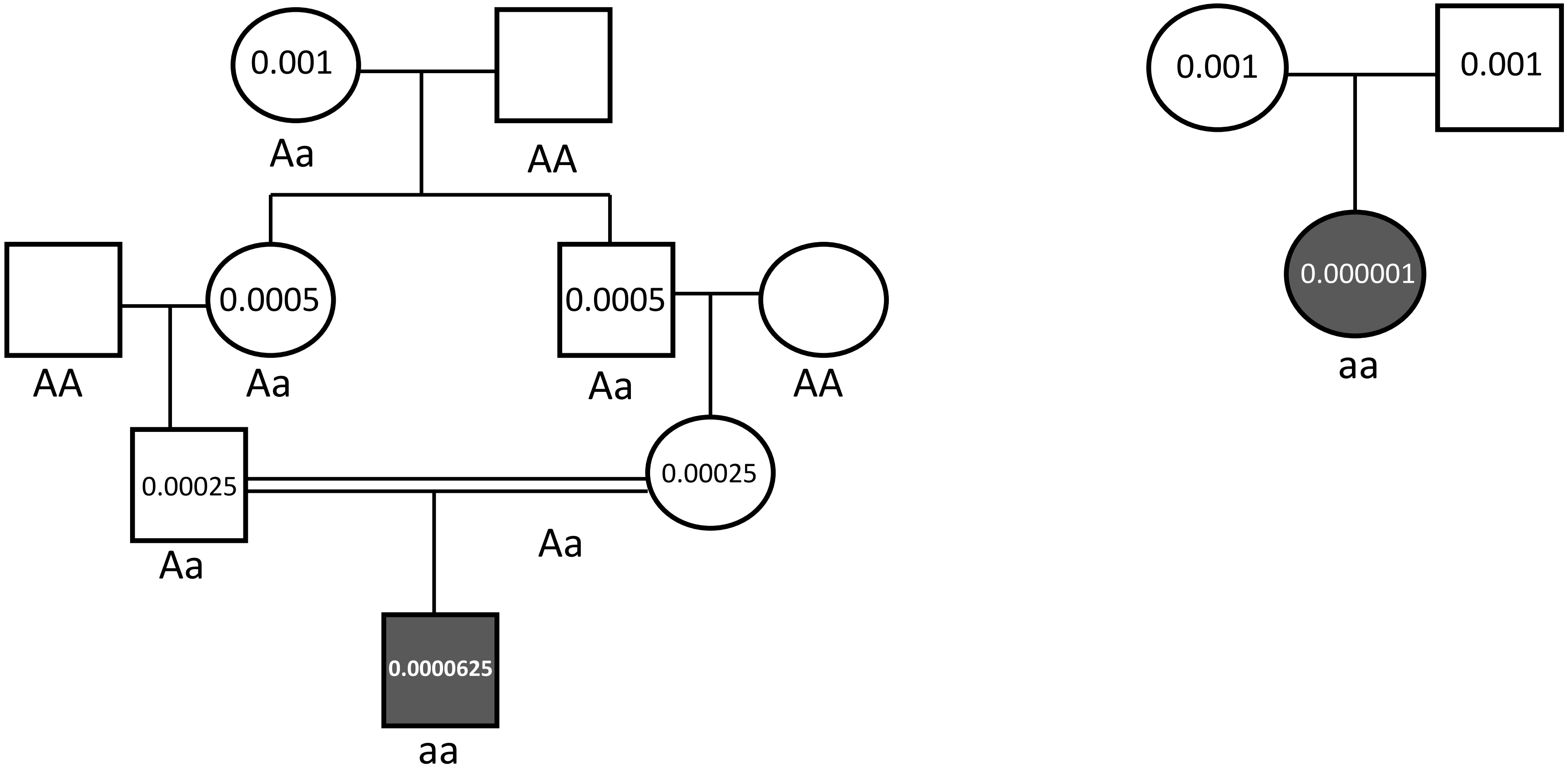

Supp. Figure S2 Comparison between offspring of outbred individuals and first cousins using the example of an allele for which q = 0.001 (frequency of 1 in thousand in a population). The true effects of consanguinity is seen in this example as there is 62.5 fold increased probability of observing a homozygote in the offspring first cousins compared to offspring of outbred individuals – even if the LoF mutation has to travel down three generations to meet its counterpart. A: wild-type allele. a: LoF allele.

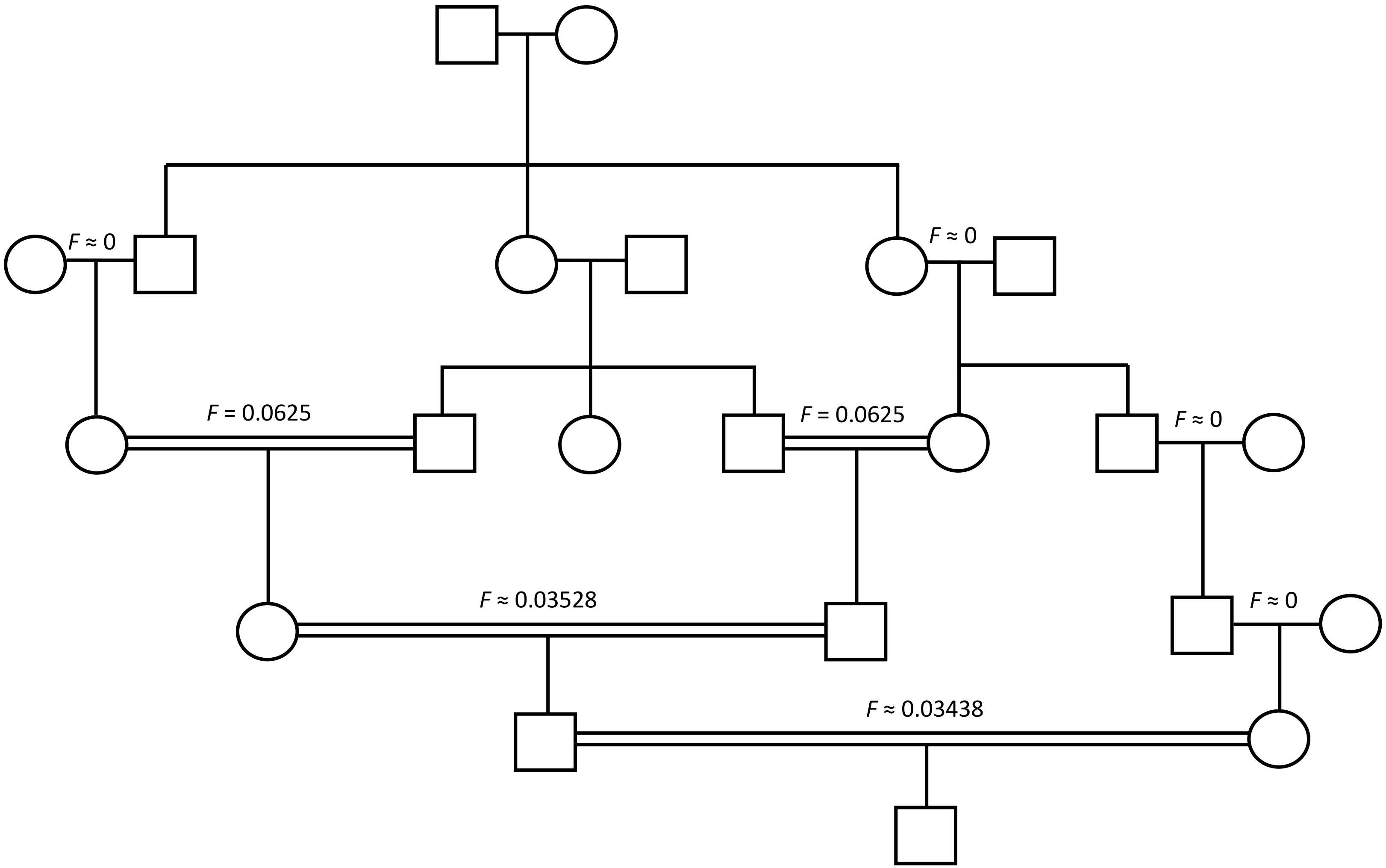

Supp. Figure S3 Example of a complex pedigree with multiple consanguineous unions. These types of complex loops of intra-familial unions which persist for many generations can harbour very large *F* values (especially if multiple uncle-niece and double first cousin unions occur). For example, even though the last consanguineous union is between second cousins ($F_{expected}$ = 0.015625), the *F* value for their offspring is 0.03438 – which translates to approximately 120% higher autozygosity compared to standard second cousin offspring. This inflation in the *F* values could also happen in endogamous populations and/or tribes. Square: Male, Circle: Female, Double lines: Consanguineous unions.

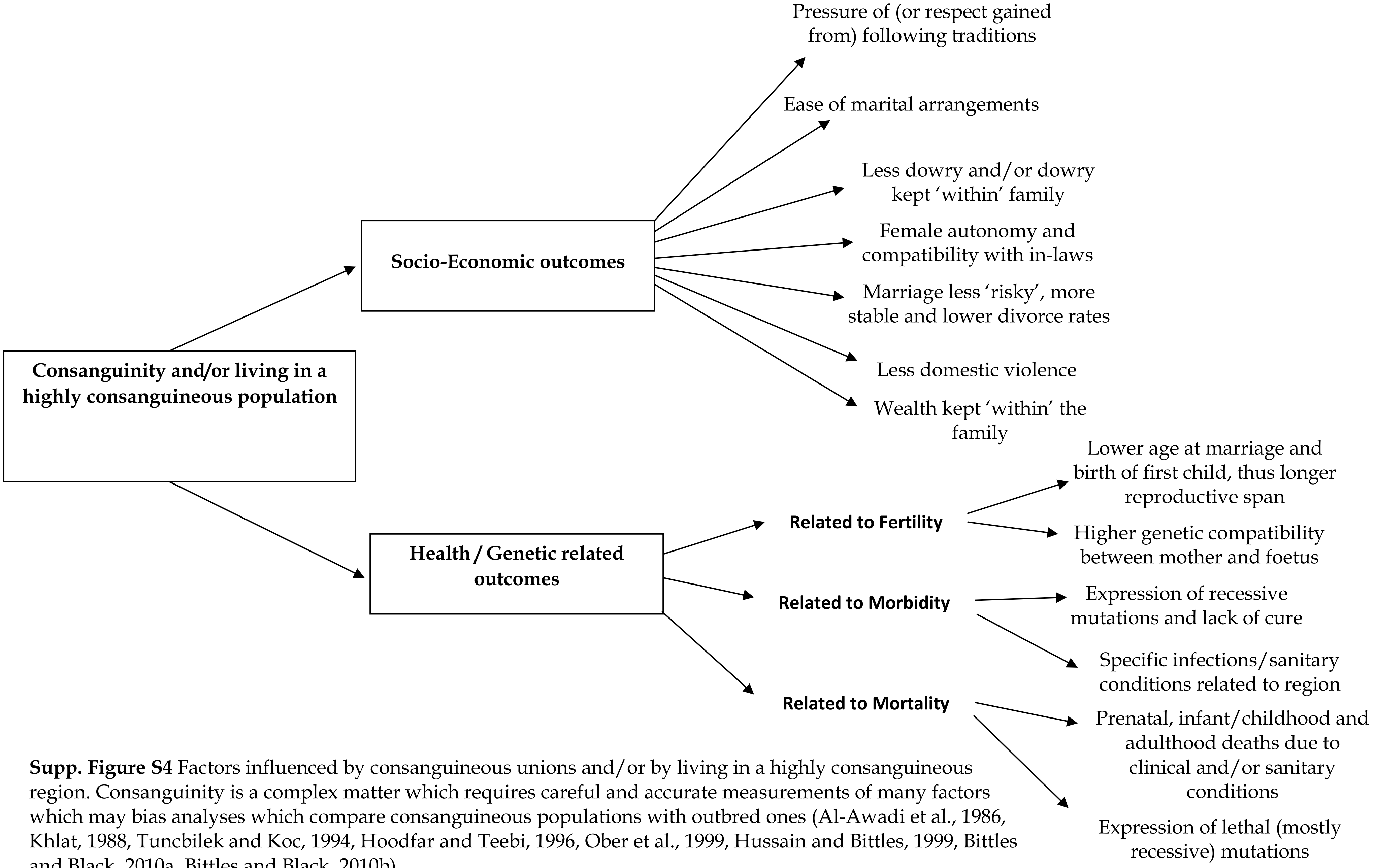

Supp. Figure S4 Factors influenced by consanguineous unions and/or by living in a highly consanguineous region. Consanguinity is a complex matter which requires careful and accurate measurements of many factors which may bias analyses which compare consanguineous populations with outbred ones (Al-Awadi et al., 1986, Khlat, 1988, Tuncbilek and Koc, 1994, Hoodfar and Teebi, 1996, Ober et al., 1999, Hussain and Bittles, 1999, Bittles and Black, 2010a, Bittles and Black, 2010b).

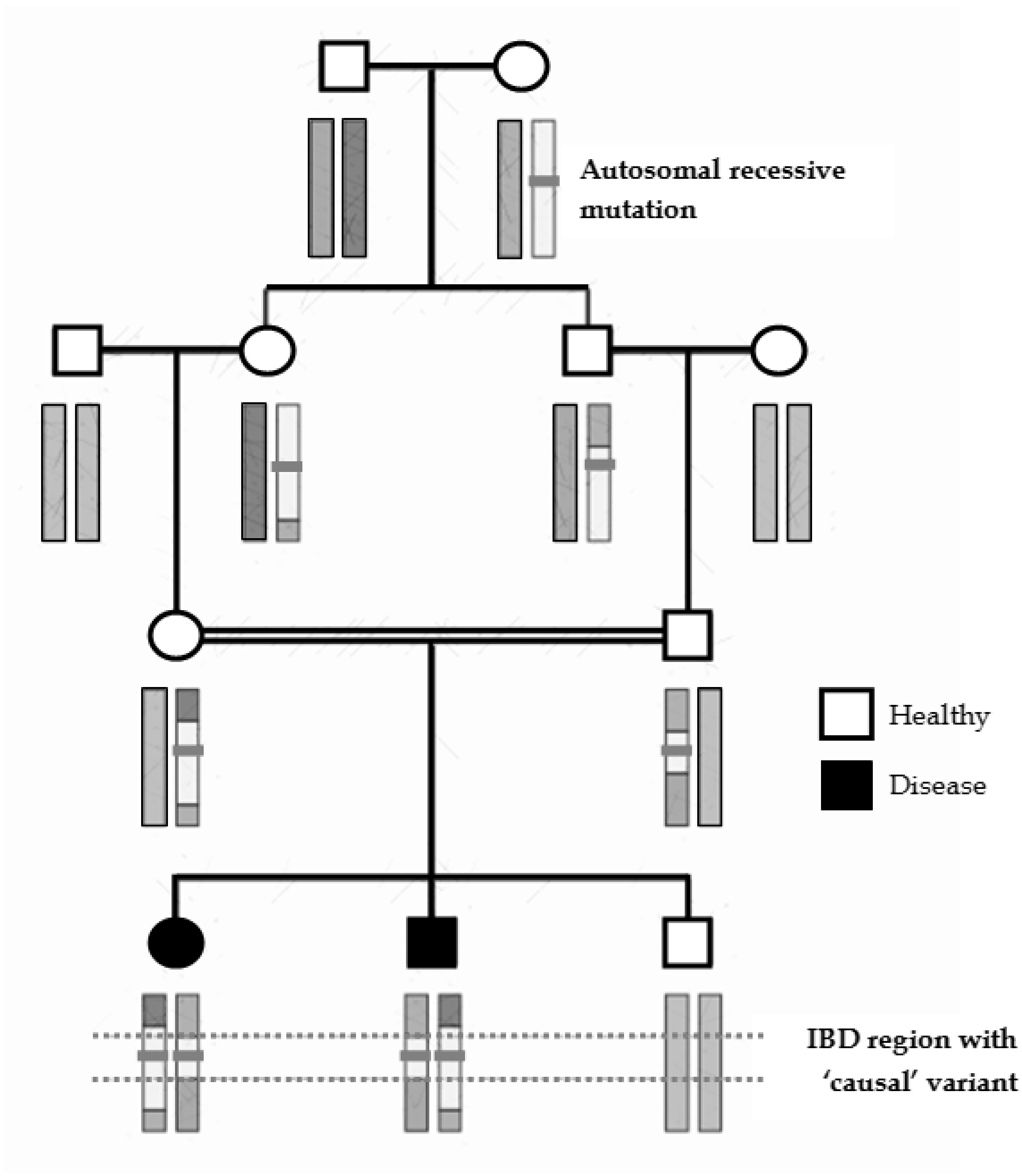

Supp. Figure S5 Autozygosity mapping and Consanguinity A recessive mutation which is inherited within an inbreeding family has the potential to be passed down the generations and be inherited in a homozygous state (due to IBD) in the offspring of the grandchildren (first cousins). Assuming familial data is available, following the autozygous regions in the generations will enable researchers to pinpoint where the causal variant is; or LRoHs can be detected. IBD: Identical by descent.

Chromosome No	Potential stop-gains	Potential missense mutations	Potential missense variants predicted 'deleterious' by SIFT	Potential missense variants predicted 'deleterious' by Polyphen-2	Potential missense variants predicted 'deleterious' by FATHMM	Potential stop-losses
**1**	487480	8467065	3347260	3854115	1221832	62572
**2**	363376	6108312	2563015	2782135	995554	44020
**3**	283426	4849265	1874572	2198890	767750	42152
**4**	205941	3388227	1297317	1485985	473172	33151
**5**	219006	3738180	1553672	1759632	504748	25432
**6**	244104	4151134	1692057	1926158	572431	28653
**7**	220408	3847838	1564136	1695240	614793	28698
**8**	168650	2881145	1151638	1265275	418389	26880
**9**	189793	3370973	1333376	1524419	474573	24432
**10**	191621	3289834	1323421	1488356	468687	24363
**11**	262358	4850828	2001340	2210778	697377	33717
**12**	253130	4361643	1745694	2001575	757405	31582
**13**	87557	1448206	549045	679773	275827	9120
**14**	155852	2672365	1020606	1182304	342163	27263
**15**	155001	2686408	1169872	1298185	452945	8352
**16**	174677	3350511	1406819	1588250	512379	12994
**17**	242866	4510502	2010220	2158614	829781	19853
**18**	72225	1223623	504444	597802	164751	4140
**19**	274257	5160678	2450500	2416241	699383	19202
**20**	107404	1984320	811427	905090	298709	14196
**21**	49159	841258	331579	373539	121269	8331
**22**	92490	1785378	729532	802734	262976	11603
**Total**	4,500,781	78,967,693	32,431,542	36,195,090	11,926,894	540,706

Supp. Table S1 Potential LoF mutations in the human genome To calculate the potential number of missense, stop gain and stop loss mutations in the human genome, we downloaded the dbNSFP database (release 2.6) and parsed the functional annotations for all potential non-synonymous single nucleotide variants. Predictions for missense variants were obtained from SIFT, Polyphen-2 and FATHMM.
